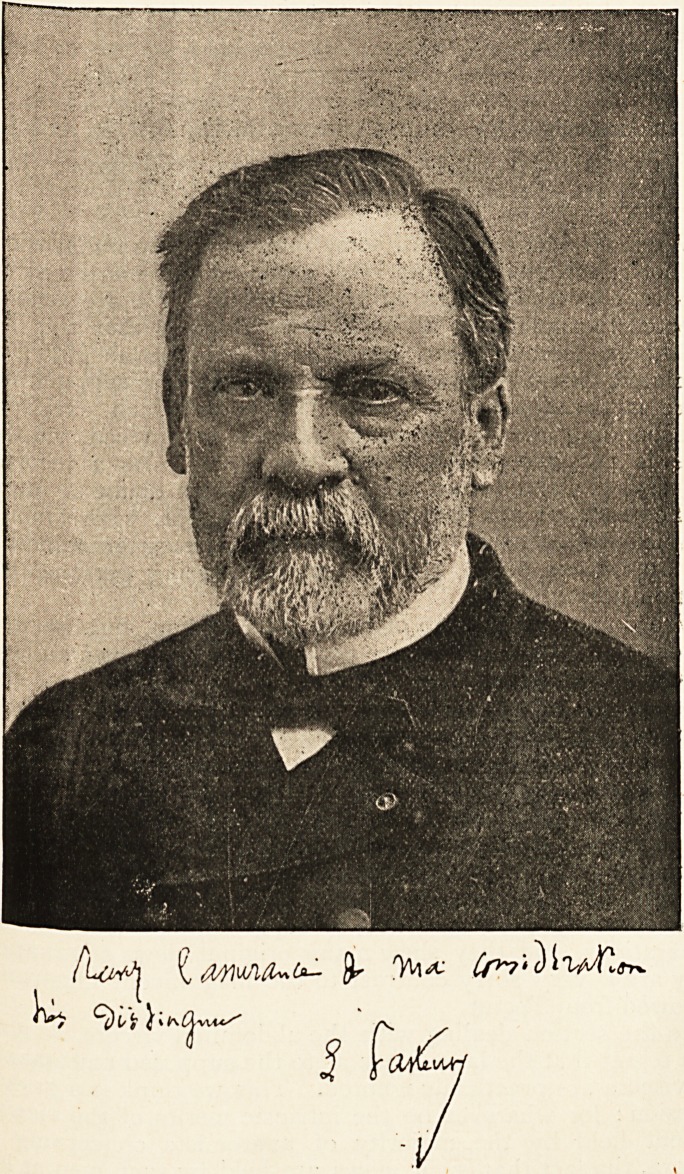# Pasteur

**Published:** 1898-06

**Authors:** 


**Pasteur.** By PERCY FRANKLAND, Ph.D., F.R.S., and Mrs. PERCY FRANKLAND. Pp. 224. London: Cassell and Company, Limited. 1898.

The Pasteur Memorial lecture delivered on March 25th, 1897, by Prof. Frankland before the Chemical Society is here reproduced, with such alterations and additions as are necessary for a less technical audience; and the result is a volume of surpassing interest, which no one unacquainted with the details of the marvellous career of Louis Pasteur can afford to leave unread.

It is a matter of congratulation that the task of compiling this brief biography was placed in the hands of one so competent to deal with both the chemical and the biological aspects of Pasteur's work as Prof. Frankland. After a few pages devoted to the subjects of parentage and birthplace, the reader is made acquainted in successive chapters with the essential features of the various lines of research so successfully exploited by the illustrious subject of the lecture. Commencing with the initial discovery of the connection of molecular structure with optical behaviour, the transfer of Pasteur's energies to the investigation of fermentation phenomena, as a consequence of the observation of the very different action of ferments upon the chemically similar substances that had hitherto occupied his attention, is lucidly described: then follow the well-known researches on vinegar, and the diseases of wine and beer, with the first excursion into the domain of animal pathology in connection with the diseases of the silkworm. From this point the investigation of infectious diseases naturally followed, and led directly to the discovery of the "vaccines" for fowl cholera and anthrax, and ultimately to the crowning achievement of all, the protective inoculation against rabies.

An account of the closing scenes of this long and laborious life, and of the present constitution of the Institut Pasteur, bring this volume of "The Century Science Series" to a close; and we feel sure that our readers will be glad to have a portrait of the distinguished subject of this memoir, which the courtesy of the editor of the *British Medical Journal* enables us to give.

The general impression left by a perusal of this appreciative sketch is that Pasteur's pre-eminence was largely due to an almost unique combination of unbounded power of speculation with an unrivalled capacity for close and accurate experimental work. Enthusiastic visionaries are plentiful: careful and painstaking investigators, if not so numerous, are to be frequently met with: but the union of the faculty for taking a comprehensive view of all possible explanations of a novel phenomenon with the power of concentrating all the mental and physical energies upon the proof or disproof of the selected theory is likely to be as rare in the future as it has been in the past. In addition, moreover, to this rare combination, Pasteur appeared on many occasions to select the correct point for experimental attack by virtue of an instinctive insight of which he could not, or at least did not, give a logical account. Whether these happy inspirations were, as suggested by Dr. Frankland, pure luck, or were the outcome of a delicate weighing of probabilities possible only to the intellect of "le grand maître," is a question for each reader to decide for himself.

## Figures and Tables

**Figure f1:**